# Chemical analysis of snus products from the United States and northern Europe

**DOI:** 10.1371/journal.pone.0227837

**Published:** 2020-01-15

**Authors:** Tameka S. Lawler, Stephen B. Stanfill, Hang T. Tran, Grace E. Lee, Patrick X. Chen, J. Brett Kimbrell, Joseph G. Lisko, Carolina Fernandez, Samuel P. Caudill, B. Rey deCastro, Clifford H. Watson

**Affiliations:** 1 Division of Laboratory Sciences, National Center for Environmental Health, Centers for Disease Control and Prevention, Atlanta, Georgia, United States of America; 2 Emory University, Rollins School of Public Health, Department of Environmental Health, Atlanta, Georgia, United States of America; 3 Oak Ridge Institute for Science and Education, Oak Ridge, Tennessee, United States of America; Medical University of South Carolina, UNITED STATES

## Abstract

**Introduction:**

Snus is an oral tobacco product that originated in Sweden. Snus products are available as fine-cut loose tobacco or in pre-portioned porous “pouches.” Some snus products undergo tobacco pasteurization during manufacturing, a process that removes or reduces nitrite-forming microbes, resulting in less tobacco-specific nitrosamine content in the product. Some tobacco companies and researchers have suggested that snus is potentially less harmful than traditional tobacco and thus a potential smoking cessation aid or an alternative to continued cigarette consumption. Although snus is available in various countries, limited information exists on snus variants from different manufacturers.

**Methods:**

Moisture, pH, nicotine, and tobacco-specific N’-nitrosamines (TSNAs) were quantified in 64 snus products made by 10 manufacturers in the United States and Northern Europe (NE). Reported means, standard errors, and differences are least-square (LS) estimates from bootstrapped mixed effects models, which accounted for correlation among repeated measurements. Minor alkaloids and select flavors were also measured.

**Results:**

Among all product types, moisture (27.4%–59.5%), pH (pH 5.87–9.10), total nicotine (6.81–20.6 mg/g, wet), unprotonated nicotine (0.083–15.7 mg/g), and total TSNAs (390–4,910 ng/g) varied widely. The LS-mean unprotonated nicotine concentration of NE portion (7.72 mg/g, SE = 0.963) and NE loose (5.06 mg/g, SE = 1.26) snus were each significantly higher than US portion snus (1.00 mg/g, SE = 1.56). Concentrations of minor alkaloids varied most among products with the highest total nicotine levels. The LS-mean NNN+NNK were higher in snus sold in the US (1360 ng/g, SE = 207) than in NE (836 ng/g, SE = 132) countries. The most abundant flavor compounds detected were pulegone, eucalyptol, and menthol.

**Conclusion:**

Physical and chemical characteristics of US and NE products labeled as snus can vary considerably and should not be considered “equivalent”. Our findings could inform public health and policy decisions pertaining to snus exposure and potential adverse health effects associated with snus.

## Introduction

Snus is a finely ground oral (smokeless) tobacco product that initially consisted of air-cured tobacco, salt, and water. The manufacturing of snus in Sweden was first mentioned in the early 1630s [1]. Over time, ingredients like humectants, alkaline agents (e.g., sodium carbonate and sodium bicarbonate), and flavorings were added to preserve the product and improve taste [2]. Snus was first introduced in the United States (US) in the 1800s and was the precursor to the most common form of smokeless tobacco, moist snuff, an unpasteurized product that contains fire-cured and fermented tobacco [1,3]. In the early 1990s, the sale of snus was banned from all European Union (EU) countries, except Sweden. Today, snus is predominately used in Northern European (NE) countries, such as Sweden, Norway, and Denmark, but is gaining popularity in other nations, like South Africa and the United States [3]. Moreover, Norway is not a part of the EU and loose snus is sold in Denmark however there is a ban on portion snus [4].

Since 2006, several snus products, including Camel, Marlboro, Triumph, and Skoal Snus, have been test marketed or released in the US market. These products are primarily sold in a pre-portioned pouch format and offered in mint and wintergreen flavors. In Sweden, snus is regulated under the Swedish Food Act and is available in a wide array of flavors (licorice, lemon, cinnamon, clove, cherry, and mint). The NE snus products may include ‘strength’ descriptors (e.g., strong, extra-strong, ultra-strong, stark, and extra-stark) on their labels and may be offered in a variety of portion sizes (e.g., mini, small, slim, medium, large, maxi). These portion size pouches, can be further divided into two categories: white and regular (original). The original portion pouch product is visually moist and brown because it undergoes an additional moisturizing process during manufacturing. For clarification, the terms portion and pouch are often used interchangeably. However, in this study for clarity these products are referred to as portion. In addition to portion snus, some NE snus products are available as loose tobacco.

For the past 30 years, Swedish Match, the largest snus manufacturer, has instituted a voluntary standard (GothiaTek®) for regulating raw materials, the manufacturing process, and the levels of certain chemicals in snus such as agricultural chemicals, benzo[a]pyrene, toxic metals, nitrite, and various tobacco-specific nitrosamines [5]. Tobacco-specific nitrosamines [TSNAs] are of public health significance because these compounds are among the most abundant and potent carcinogens in tobacco products [3]. Because microorganisms are capable of producing nitrite, which contributes to TSNA formation, the production of snus often incorporates heat treatment (pasteurization) to kill microorganisms [6–8]. From 1983 to 2004, systemic measures to minimize nitrosamines in snus has resulted in an 85% decrease in TSNA levels in Swedish snus, made under GothiaTek® standards [9–10]. In 2014, the combined concentration of two carcinogenic TSNAs, N'-nitrosonornicotine (NNN) and 4-(methylnitrosamino)-1-(3-pyridyl)-1-butanone (NNK), in snus made by Swedish Match averaged 0.49 μg/g, which was well below the 0.95 μg/g GothiaTek® threshold and levels reported in prior studies [1,3,11–12].Because of its lowered TSNA levels, snus is often perceived as a less harmful form of tobacco when compared with cigarette smoking [13–14]. However, further investigation into potential lifetime nicotine dependence and other health risks [15–17] associated with the use of snus is warranted to inform tobacco policies and effective public health messages about these products.

This study reports the moisture, pH, and levels of total nicotine, unprotonated nicotine, alkaloids, TSNAs, and flavor compounds in 64 snus products made by ten manufacturers in Northern Europe (n = 56) and the United States (n = 8). At present, it is not known if the chemical composition of snus products made in these countries is similar. This study was designed to generate data enabling comparisons of snus with other tobacco products, and to determine whether US and NE snus products differ chemically. Study results may provide valuable information to snus consumers, clinicians, tobacco regulators, and policymakers.

## Materials and methods

### Sample collection

From 2013–2014, a convenience sample of 64 (56 NE and 8 US) snus products were purchased through Lab Depot (Dawsonville, GA) from stores in the Atlanta, Georgia area or on the internet. Upon receipt, smokeless samples were logged into a custom database, assigned barcodes with a unique ID, and stored at– 70 ^o^C until analyzed.

### Tobacco analysis

#### Measurement of moisture and pH content

Total moisture content was measured using a methodology described elsewhere [12]. The pH values were determined in a 1-g sample of each product in a 10-mL aliquot of distilled, deionized water using a Sirius Vinotrate pH meter (Sirius Analytical Ltd., East Sussex, United Kingdom) calibrated at pH 4.01 and 7.00. Measurements for pH at 5-, 15-, 30-, and 60-min intervals were averaged. The pH protocol used is fully described elsewhere [12]. Averages of duplicate measures for moisture and pH measurements are reported.

#### Quantification of nicotine by GC-MS

Total nicotine concentrations were measured using a gas chromatography-mass spectrometry (GC-MS) method as described previously [18]. Briefly, approximately 1 g of tobacco was extracted with 50 mL of MTBE extraction solution (containing quinoline internal standard) and 5 mL of 2N NaOH. Samples were shaken for 2 hours, and a 1-μL aliquot was analyzed by GC-MS using selected ion monitoring (SIM). Nicotine measurements for all products were performed in triplicate (n = 3).

#### Quantification of minor alkaloids by GC-MS/MS

Five minor alkaloids (nornicotine, myosmine, anatabine, anabasine, and isonicoteine) were measured by gas chromatography-triple quadrupole mass spectrometry (GC-MS/MS) in multiple reaction monitoring (MRM) mode in triplicate [19].

#### Quantification of tobacco-specific N-nitrosamines by LC-MS/MS

Each product was analyzed in triplicate for five tobacco-specific-N’-nitrosamine compounds: *N*′-nitrosonornicotine (NNN), 4-(methylnitrosamino)-1-(3-pyridyl)-1-butanone (NNK), *N*′-nitrosoanatabine (NAT), *N*′-nitrosoanabasine (NAB), and 4-(methylnitrosamino)-1-(3-pyridyl)-1-butanol (NNAL). Snus samples were weighed to approximately 0.25 g, in an amber vial, and spiked with ^13^C-labeled internal standards. Samples were then extracted with 10 mL of aqueous ammonium acetate buffer, shaken for one hour at 250 rpm, and filtered with a 0.45 μm PVDF syringe filter. An Agilent 1100 high-performance liquid chromatography (HPLC) system (Agilent Corporation, Santa Clara, California) fitted with an Xterra MS C18 column (4.6 x 50mm, 5μm) provided well resolved chromatographic peaks (Waters Corporation, Milford, MA). The HPLC gradient was comprised of eluent A, 5 mM ammonium acetate solution; eluent B was acetonitrile. The column temperature was constant at 60°C with a flow rate of 1.0 mL/min. The initial organic phase concentration was held at 5% for 1 minute then increased to 35% after two minutes. This 35% component B plateau was held for 3 min, then decreased to 5% and held for 3 min for column equilibration. The Triple Quad 5500 (AB Sciex, Framingham, MA) was operated in electrospray, positive polarity using multiple reaction monitoring (MRM) mode, and AB Sciex Analyst 1.6 software was utilized to process and integrate the reconstructed ion chromatograms.

#### Quantification of ten flavor compounds by GC-MS

Concentrations of eucalyptol, camphor, menthol, pulegone, ethyl salicylate, methyl salicylate, cinnamaldehyde, eugenol, diphenyl ether, and coumarin were measured in triplicate using GC-MS in selected ion monitoring (SIM) and expressed in μg/g. The analytical methodology is reported elsewhere [20].

### Statistical analysis

Moisture [percent], pH, nicotine [mg/g], and TSNAs [ng/g] were compared among snus products categorized by region (Northern Europe or United States), product type (portion or loose), portion type (white or original), and by strength descriptor as indicated on the package label. We combined products whose labels included descriptors of ‘strength’ (e.g., strong, extra-strong, ultra-strong, stark, and extra-stark) into one category: “strong.” Products without strength descriptors were categorized as “standard.” For each of several snus products within a product type, measurements of moisture, pH, and unprotonated nicotine, were repeated in duplicate, and in triplicate for total nicotine, NAB, NAT, NNK, NNN, NNN+NNK, NNAL, and total TSNA. In order to represent the correlation among the repeated measurements, mixed effects models were configured with random effects indicating snus product nested within manufacturer and region, manufacturer nested within region, as well as random intercepts. Fixed effects represented combinations of product characteristics: region, product type, portion type, and ‘strength’. Because measurement distributions were not entirely compatible with assumptions underlying calculation of means and standard errors, the least-square statistics were derived from analyses of 10,000 bootstrap resamples of the data, stratified by region, manufacturer, and snus product. Statistical calculations were conducted using the SURVEYSELECT, MIXED, MEANS, and UNIVARIATE subroutines of the SAS^®^ (v. 9.4) statistical software application. Statistical significance was set at α ≤ 0.05. Tabulated means, standard errors, and differences are bootstrapped least-square (LS) estimates from these mixed effects models (Tables 1 and 2). Mixed effects models were not applied to statistics displayed in Figures, nor statistics reported for alkaloids and flavors.

**Table 1:** Least-square means of moisture, pH, and nicotine concentrations for snus categorized by region and product type.

Comparison	Products		Moisture^d^ (%)	pH^d^	Total Nicotine^e^ (mg/g, wet)	Unprotonated^d^ Nicotine (%)	Unprotonated^d^ Nicotine (mg/g, wet)
**A**	**NORTHERN EUROPEAN SNUS**						
	**Portion (Standard Descriptors)**	**LS-mean (SE)** ^a^	**45.3 (1.83)**	**8.08 (0.232)**	**11.5 (0.907)**	**49.8 (7.95)**	**5.61 (0.969)**
	**(n = 33)**	LS Difference (SE) ^b^	Ref. A^**f**^	Ref.A	Ref.A	Ref.A	Ref.A
		Difference, p-Value	–	–	–	–	–
		Range ^c^	27.4–57.4	6.94–9.10	7.25–16.4	7.68–92.3	1.00–12.7
	**Loose (Standard Descriptors)** ^**g**^	**LS-mean (SE)**	**51.8 (2.65)**	**7.53 (0.267)**	**11.5 (1.25)**	**27.5 (9.99)**	**2.94 (1.22)**
	**(n = 6)**	LS Difference (SE)	6.51 (2.18)	-0.450 (0.150)	-0.0477 (0.986)	-22.3 (6.91)	-2.67 (0.847)
		Difference, p-Value	0.0043	0.0042	0.9616	0.0022	0.0027
		Range	48.5–59.5	7.19–8.18	6.81–20.6	12.8–58.8	1.78–5.57
	**Portion (Strong Descriptors)**	**LS-mean (SE)**	**45.3 (2.16)**	**8.18 (0.246)**	**16.6 (1.04)**	**60.1 (8.76)**	**9.84 (1.07)**
	**(n = 16)**	LS Difference (SE)	-0.0175 (1.73)	0.202 (0.119)	5.09 (0.781)	10.3 (5.49)	4.23 (0.673)
		Difference, p-Value	0.9919	0.0972	< .0001	0.0666	< .0001
		Range	37.6–55.3	7.88–8.65	10.9–20.6	42.0–81.0	5.90–15.7
	**US SNUS**						
	**Portion (Standard Descriptors)**	**LS-mean (SE)**	**30.8 (3.05)**	**6.62 (0.366)**	**10.8 (1.49)**	**11.1 (12.8)**	**1.00 (1.56)**
	**(n = 8)**	LS Difference (SE)	-14.5 (3.56)	-1.35 (0.434)	-0.700 (1.75)	-38.7 (15.1)	-4.60 (1.84)
		Difference, p-Value	0.0016	0.0142	0.6965	0.0344	0.0351
		Range	28.2–33.0	5.87–7.66	8.02–13.6	0.708–30.6	0.083–2.85
**B**	**NORTHERN EUROPEAN SNUS**						
	**Portion (White)**	**LS-mean (SE)**	**44.6 (2.09)**	**8.09 (0.242)**	**14.2 (1.01)**	**55.8 (8.54)**	**7.72 (1.04)**
	**(n = 28)**	LS Difference (SE)	Ref.B^**f**^	Ref.B	Ref.B	Ref.B	Ref.B
		Difference, p-Value	–	–	–	–	–
		Range	27.4–57.4	6.94–9.10	7.25–16.9	7.68–92.3	1.26–12.7
	**Portion (Original)**	**LS-mean (SE)**	**45.9 (1.95)**	**8.07 (0.238)**	**13.9 (0.957)**	**54.2 (8.27)**	**7.72 (1.01)**
	**(n = 21)**	LS Difference (SE)	1.30 (1.81)	-0.0216 (0.125)	-0.232 (0.817)	-1.65 (5.76)	-0.0061 (0.705)
		Difference, p-Value	0.4740	0.8636	0.7773	0.7759	0.9931
		Range	37.6–52.8	7.12–8.92	7.88–20.6	11.2–88.8	1.00–15.7
**C**	**NORTHERN EUROPEAN SNUS**						
	**Portion (All)**	**LS-mean (SE)**	**45.3 (1.81)**	**8.08 (0.232)**	**14.1 (0.896)**	**55.0 (7.90)**	**7.72 (0.963)**
	**(n = 49)**	LS Difference (SE)	Ref.C^**f**^	Ref.C	Ref.C	Ref.C	Ref.C
		Difference, p-Value	–	–	–	–	–
		Range	27.4–57.4	6.94–9.10	7.25–20.6	7.68–92.3	1.00–15.7
	**Loose (All)** ^**h**^	**LS-mean (SE)**	**51.8 (2.75)**	**7.63 (0.272)**	**14.0 (1.30)**	**32.7 (10.3)**	**5.06 (1.26)**
	**(n = 7)**	LS Difference (SE)	6.51 (2.18)	-0.450 (0.150)	-0.0477 (0.986)	-22.3 (6.91)	-2.67 (0.847)
		Difference, p-Value Range	0.0043 48.5–59.5	0.0042 7.19–8.18	0.9616 6.81–20.6	0.0022 12.8–58.8	0.0027 1.78–6.19
	**US SNUS**						
	**Portion (All)**	**LS-mean (SE)**	**30.8 (3.05)**	**6.62 (0.366)**	**10.8 (1.49)**	**11.2 (12.8)**	**1.00 (1.56)**
	**(n = 8)**	LS Difference (SE)	-14.5 (3.56)	-1.35 (0.434)	-0.700 (1.75)	-38.7 (15.1)	-4.60 (1.84)
		Difference, p-Value	0.0016	0.0142	0.6965	0.0344	0.0351
		Range	28.2–33.0	5.87–7.66	8.02–13.6	0.708–30.6	0.083–2.85
**D**	**NORTHERN EUROPEAN SNUS**						
	**All (Portion and Loose)**	**LS-mean (SE)**	**47.4 (1.91)**	**7.93 (0.236)**	**14.0 (0.938)**	**47.5 (8.14)**	**6.83 (0.992)**
	**(n = 56)**	LS Difference (SE)	Ref. D^**f**^	Ref.D	Ref.D	Ref.D	Ref.D
		Difference, p-Value	–	–	–	–	–
		Range	27.4–59.5	6.94–9.10	6.81–20.6	7.68–92.3	1.00–15.7
	**US SNUS**						
	**All (Portion)**	**LS-mean (SE)**	**30.8 (3.05)**	**6.62 (0.366)**	**10.8 (1.49)**	**11.2 (12.8)**	**1.00 (1.56)**
	**(n = 8)**	LS Difference (SE)	-16.6 (3.60)	-1.30 (0.436)	-3.23 (1.76)	-36.4 (15.2)	-5.83 (1.85)
		Difference, p-Value	0.0006	0.0169	0.0935	0.0439	0.0123
		Range	28.2–33.0	5.87–7.66	8.02–13.6	0.708–30.6	0.083–2.85

Table 1. Differences in means are evaluated in four comparisons with: A) Northern European portion standard snus; B) Northern European portion white snus; C) Northern European portion snus; and D) Northern European portion and loose snus.

Note.

^**a**^ LS-mean (SE). Least-square mean (standard error). Means, standard errors, and differences are least-square estimates from mixed effects models.

^**b**^ Difference in least-square means.

^**c**^ Range. These concentrations were identified among all replicate measurements for the region and snus product category.

^**d**^ n = 2 for each snus product for measurement of moisture, pH, and unprotonated nicotine. Means, standard errors, and differences are least-square estimates from mixed effects models of 10,000 bootstrap resamples of the data.

^**e**^ n = 3 for each snus product for measurement of total nicotine. Means, standard errors, and differences are least-square estimates from mixed effects models of 10,000 bootstrap resamples of the data.

^**f**^ Ref., reference product. The least-square mean of this product was subtracted from other product types in each category to compute the difference. Ref.A is Northern Europe Portion Standard. Ref.B is Northern Europe Portion White. Ref.C is Northern Europe Portion. Ref.D is Northern Europe All.

^**g**^ One loose product labeled with a “strong” descriptor was excluded from the analysis.

^**h**^ Loose (All) includes 1 product labeled with a “strong” descriptor and 6 products with “standard” descriptors.

**Table 2:** Least-square means of five tobacco-specific N’-nitrosamines in snus categorized by region and product type.

Comparison	Products		NAT	NAB	NNN	NNK	NNAL	NNN+NNK	Total TSNAs
			ng/g, wet; n = 3 ^d^						
**A**	**NORTHERN EUROPEAN SNUS Portion (Standard)**	**LS-mean (SE)** ^a^	**405 (136)**	**41.9 (11.4)**	**619 (105)**	**173 (41.6)**	**24.2 (4.17)**	**792 (129)**	**1260 (241)**
	**(n = 33)**	LS Difference (SE) ^b^	Ref. A^**e**^	Ref.A	Ref.A	Ref.A	Ref.A	Ref.A	Ref.A
		Difference, p-Value	–	–	–	–	–	–	–
		Range ^c^	145–741	7.82–157	251–1040	50.7–431	7.09–52.3	316–1250	501–2060
	**Loose (Standard)**	**LS-mean (SE)**	**299 (157)**	**26.9 (12.2)**	**464 (131)**	**109 (56.1)**	**18.3 (4.63)**	**566 (166)**	**918 (300)**
	**(n = 6)**	LS Difference (SE)	-106 (89.5)	-15.1 (4.86)	-155 (89.7)	-64.2 (42.9)	-6.12 (2.37)	-192 (122)	-344 (203)
		Difference, p-Value	0.2425	0.0032	0.0894	0.1408	0.0126	0.8496	0.0965
		Range	80.3–606	6.18–51.6	193–823	45.1–172	4.88–25.5	244–987	390–1660
	**Portion (Strong)**	**LS-mean (SE)**	**733 (144)**	**47.5** ^**f**^ **(11.8)**	**714 (115)**	**311 (47.4)**	**24.1 (4.32)**	**1030 (142)**	**1820 (264)**
	**(n = 16)**	LS Difference (SE)	328 (71.3)	5.57 (4.13)	94.4 (71.3)	138 (34.1)	-0.302 (1.89)	233 (89.6)	561 (162)
		Difference, p-Value	< .0001	0.1820	0.1912	0.0002	0.8738	0.0118	0.0010
		Range	275–2210	9.61–92.1	49.5–1930	41.9–696	3.88–40.0	403–2600	758–4910
	**US SNUS**								
	**Portion (Standard)**	**LS-mean (SE)**	**804 (214)**	**62.9 (17.7)**	**1050 (168)**	**321 (68.1)**	**23.1 (6.46)**	**1360 (207)**	**2220 (386)**
	**(n = 8)**	LS Difference (SE)	399 (254)	21.0 (21.1)	428 (198)	148 (79.8)	-1.35 (7.67)	569 (244)	958 (455)
		Difference, p-Value	0.1615	0.3478	0.0628	0.0933	0.8650	0.0445	0.0699
		Range	349–1820	18.6–109	502–1600	145–572	9.44–46.1	695–1840	1140–3720
**B**	**NORTHERN EUROPEAN SNUS**								
	**Portion (White)**	**LS-mean (SE)**	**545 (142)**	**44.1** ^**g**^ **(11.7)**	**618 (112)**	**276 (46.0)**	**25.3 (4.27)**	**894 (139)**	**1510 (258)**
	**(n = 28)**	LS Difference (SE)	Ref.B^**e**^	Ref.B	Ref.B	Ref.B	Ref.B	Ref.B	Ref.B
		Difference, p-Value	–	–	–	–	–	–	–
		Range	145–614	7.82–157	49.5–915	50.7–518	4.09–52.3	316–1200	501–1860
	**Portion (Original)**	**LS-mean (SE)**	**593 (139)**	**45.4** ^**h**^ **(11.6)**	**715 (109)**	**208 (43.8)**	**23.2 (4.21)**	**923 (134)**	**1580 (250)**
	**(n = 21)**	LS Difference (SE)	48.3 (74.8)	1.29 (4.29)	97.3 (74.7)	-68 (35.6)	-2.13 (1.98)	28.3 (93.9)	75 (170)
		Difference, p-Value	0.5206	0.7598	0.1983	0.0616	0.2869	0.7649	0.6603
		Range	184–2210	9.10–98.1	300–1930	41.9–696	3.88–42.2	358–2600	574–4910
**C**	**NORTHERN EUROPEAN SNUS**								
	**Portion (All)**	**LS-mean (SE)**	**569 (136)**	**44.7** ^**f**^**(11.4)**	**666 (104)**	**242 (41.2)**	**24.3 (4.12)**	**909 (128)**	**1540 (239)**
	**(n = 49)**	LS Difference (SE)	Ref.C^**e**^	Ref.C	Ref.C	Ref.C	Ref.C	Ref.C	Ref.C
		Difference, p-Value	–	–	–	–	–	–	–
		Range	145–2210	7.82–157	49.5–1930	41.9–696	3.88–52.3	316–2600	501–4910
	**Loose (All)**^**i**^	**LS-mean (SE)**	**463 (160)**	**29.7 (12.3)**	**511 (134)**	**178 (58.0)**	**18.2 (4.70)**	**690 (167)**	**1200 (308)**
	**(n = 7)**	LS Difference (SE)	-106 (89.5)	-15.1 (4.86)	-155 (89.7)	-64.2 (42.9)	-6.12 (2.37)	-219 (113)	-344 (203)
		Difference, p-Value	0.2425	0.0032	0.0894	0.1408	0.0126	0.0575	0.0965
		Range	80.3–606	6.18–51.6	193–823	37.4–172	3.14–25.5	244–987	390–1660
	**US SNUS**								
	**Portion (All)**	**LS-mean (SE)**	**804 (214)**	**62.9 (17.8)**	**1050 (168)**	**321 (68.1)**	**23.1 (6.46)**	**1360 (207)**	**2220 (386)**
	**(n = 8)**	LS Difference (SE)	259 (254)	19.0 (21.2)	428 (198)	148 (79.8)	-1.35 (7.67)	569 (244)	958 (455)
		Difference, p-Value	1.0000	1.0000	0.0628	0.0933	0.8650	0.0445	0.0699
		Range	349–1820	18.6–109	502–1600	145–572	9.44–46.1	695–1840	1140–3720
**D**	**NORTHERN EUROPEAN SNUS**								
	**All (Portion and Loose)**	**LS-mean (SE)**	**534 (138)**	**39.7** ^**f**^ **(11.5)**	**615 (107)**	**220 (42.9)**	**22.2 (4.18)**	**836 (132)**	**1430 (246)**
	**(n = 56)**	LS Difference (SE)	Ref.D^**e**^	Ref.D	Ref.D	Ref.D	Ref.D	Ref.D	Ref.D
		Difference, p-Value	–	–	–	–	–	–	–
		Range	80.3–2210	6.18–157	49.5–1930	37.4–696	3.14–52.3	244–2600	390–4910
	**US SNUS**								
	**All (Portion)**	**LS-mean (SE)**	**804 (214)**	**62.9 (17.7)**	**1050 (168)**	**321 (68.1)**	**23.1 (6.46)**	**1360 (207)**	**2220 (386)**
	**(n = 8)**	LS Difference (SE)	270 (255)	23.2 (21.1)	432 (199)	101 (80.5)	0.840 (7.70)	525 (246)	793 (458)
		Difference, p-Value	0.3260	0.3024	0.0613	0.2392	0.9145	0.0605	0.1229
		Range	349–1820	18.6–109	502–1600	145–572	9.44–46.1	695–1840	1140–3720

Table 2. Differences in means are evaluated in four comparisons with: A) Northern European portion standard snus; B) Northern European portion white snus; C) Northern European portion snus; and D) Northern European portion and loose snus.

Note.

^**a**^ LS-mean (SE). Least-square mean (standard error). Means, standard errors, and differences are least-square estimates from mixed effects models.

^**b**^ Difference in least-square means.

^**c**^ Range. These levels were identified among all replicate measurements for the region and snus product category.

^**d**^ n = 3 for each snus product for measurement of NAT, NAB, NNN, NNK, and NNAL. Means, standard errors, and differences are least-square estimates from mixed effects models of 10,000 bootstrap resamples of the data.

^**e**^ Ref., reference product. The least-square mean of this product was subtracted from other product types in each category to compute the difference. Ref.A is Northern Europe Portion Standard. Ref.B is Northern Europe Portion White. Ref.C is Northern Europe Portion. Ref.D is Northern Europe All.

^**f**^ NAB levels were non-detected in three products.

^**g**^ One product had non-detected NAB levels.

^**h**^ Two products had non-detected NAB levels.

^**i**^ Loose (All) includes 1 product labeled with a “strong” descriptor and 6 products with “standard” descriptors.

## Results

We examined 64 snus products, including loose and portioned products, where 56 products were from Northern Europe (NE) and 8 from the United States (US). Of the 64 products, 57 (89%) were portion snus (49 NE and 8 US) and seven products (11%) were loose snus (all of which were from Northern Europe). All eight US snus products investigated in this study were tobacco portion style. The average weight per portion for both regions was approximately 0.85 g. The portion weight or size refers to the mass of tobacco and portion material per serving. On average, US portion snus ranged from 0.59–1.11 g and European portion snus ranged from 0.33–1.13 g. Among all analytes, measurements encompassed broad ranges: moisture, 27.4% to 59.5%; total nicotine, 6.81 to 20.6 mg/g, wet; and pH, 5.87 to 9.10 corresponding to 0.71% and 92.3% unprotonated nicotine, respectively.

Least-square means (LS-means) of moisture, pH, and nicotine concentrations for NE and US snus and subcategories are summarized in Table 1. The LS-mean pH for NE snus was 7.93 (SE = 0.236) and significantly higher compared to pH 6.62 (SE = 0.366) for US snus (Table 1 –Comparison D). The total nicotine concentrations of 16 of 56 NE snus products were higher (range 13.7–20.6 mg/g) than all products from the US (range 8.02–13.6 mg/g). Unprotonated nicotine concentrations, calculated using pH and total nicotine concentrations, varied widely among all products, ranging from 0.083 to 15.7 mg/g (189-fold range across brands). Among NE snus, 82% (46 of 56) of unprotonated nicotine concentrations (range 3.14–15.7 mg/g) were higher than all US products (range 0.083–2.85 mg/g). In Table 1 –Comparison A, unprotonated nicotine concentrations were significantly higher among NE portion standard snus compared to US portion standard snus (5.61 [SE = 0.969] vs. 1.00 [SE = 1.56] mg/g, wet). Additionally, among the 33 NE portion snus products labeled without strength descriptors (referred to as “standard”), the LS-mean total and unprotonated nicotine concentrations were 11.5 (SE = 0.907) and 5.61 (SE = 0.969) mg/g, wet, respectively. The LS-mean total and unprotonated nicotine concentrations of 16 NE portion products labeled with “strength” descriptors on their packaging (referred henceforth as “strong”), were 16.6 (SE = 1.04) and 9.84 (SE = 1.07) mg/g, wet, respectively. Both total nicotine and unprotonated nicotine concentrations were significantly higher among NE snus labeled “strong” compared to snus with “standard” descriptors (11.5 [SE = 0.907] vs. 16.6 [SE = 1.04] mg/g wet and 5.61 [SE = 0.969] vs. 9.84 [SE = 1.07] mg/g wet). In Table 1 –Comparison B, there were no detectable differences in moisture, pH, and nicotine concentrations between NE white and original portion categories.

Table 1 –Comparison C shows LS-mean unprotonated nicotine concentrations for both NE portion (7.72 mg/g [SE = 0.963]) and NE loose (5.06 mg/g [SE = 1.26]) snus were each significantly higher than US portion snus (1.00 mg/g [SE = 1.56]). Among NE portion snus, 43 of the 49 portion products had higher LS-mean unprotonated nicotine levels than US portion snus. The LS-mean unprotonated nicotine levels of all 7 NE loose snus products varied more than 3-fold across brands. Moreover, among NE portion and NE loose snus categories there were statistically significant differences in the LS-mean moisture (45.3 [SE = 1.81] vs. 51.8 [SE = 2.75] percent), pH (8.08 [SE = 0.232] vs. 7.63 [SE = 0.272]), and unprotonated nicotine (7.72 [SE = 0.963] vs. 5.06 [SE = 1.26] mg/g wet) concentrations. Overall, the LS-mean unprotonated nicotine concentration of NE loose snus was five times more than that of US portion products but lower than the LS-mean of NE portion varieties.

**Fig 1:**
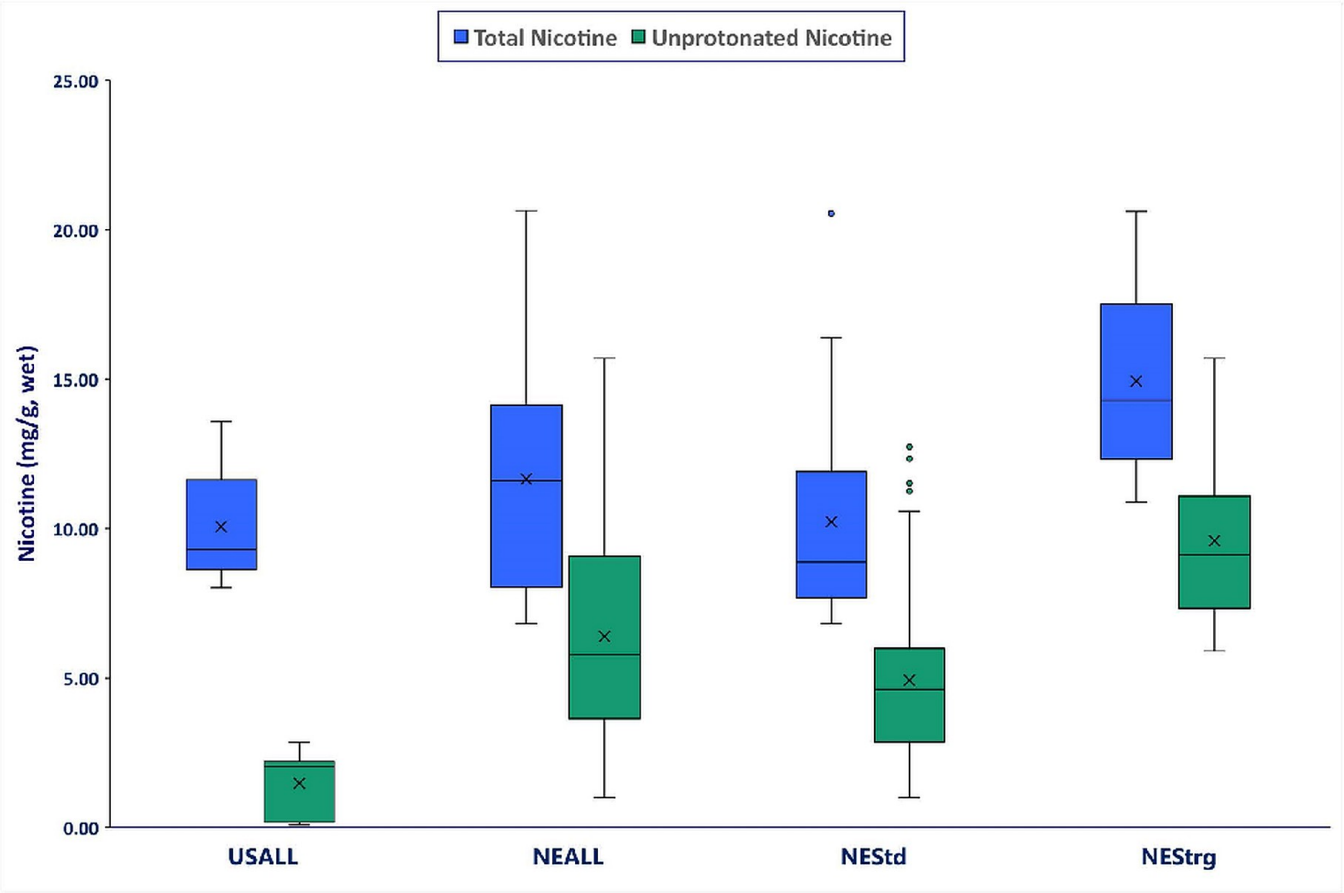
Total and unprotonated nicotine concentrations (mg/g, wet weight) for all US snus (USALL), all Northern Europe snus (NEALL), all NE snus with “standard” descriptors (NEStd) and all NE snus with “strong” descriptors (NEStrg). Note: USALL (n = 8: all 8 portion); NEALL (n = 56: 7 loose and 49 portion); NEStd. (n = 39: 6 loose and 33 portion); NEStrg (n = 17: 1 loose and 16 portion).

The highest total and unprotonated nicotine concentrations corresponded to all 17 products (16 NE portion and 1 NE loose) with “strong” descriptors on their packaging comprising thirty percent of NE snus products. For products in the “strong” descriptor category (Fig 1), the mean total nicotine and unprotonated nicotine concentrations were 14.9 and 9.59 mg/g, respectively. In products with “standard” descriptors [n = 39 (33 NE portion and 6 NE loose)], the mean total and unprotonated concentrations were 10.2 and 4.93 mg/g, respectively. NE products with “strong” descriptors had approximately 46% and 94% higher concentrations of total nicotine and unprotonated nicotine, respectively, than products with “standard” descriptors.

**Fig 2:**
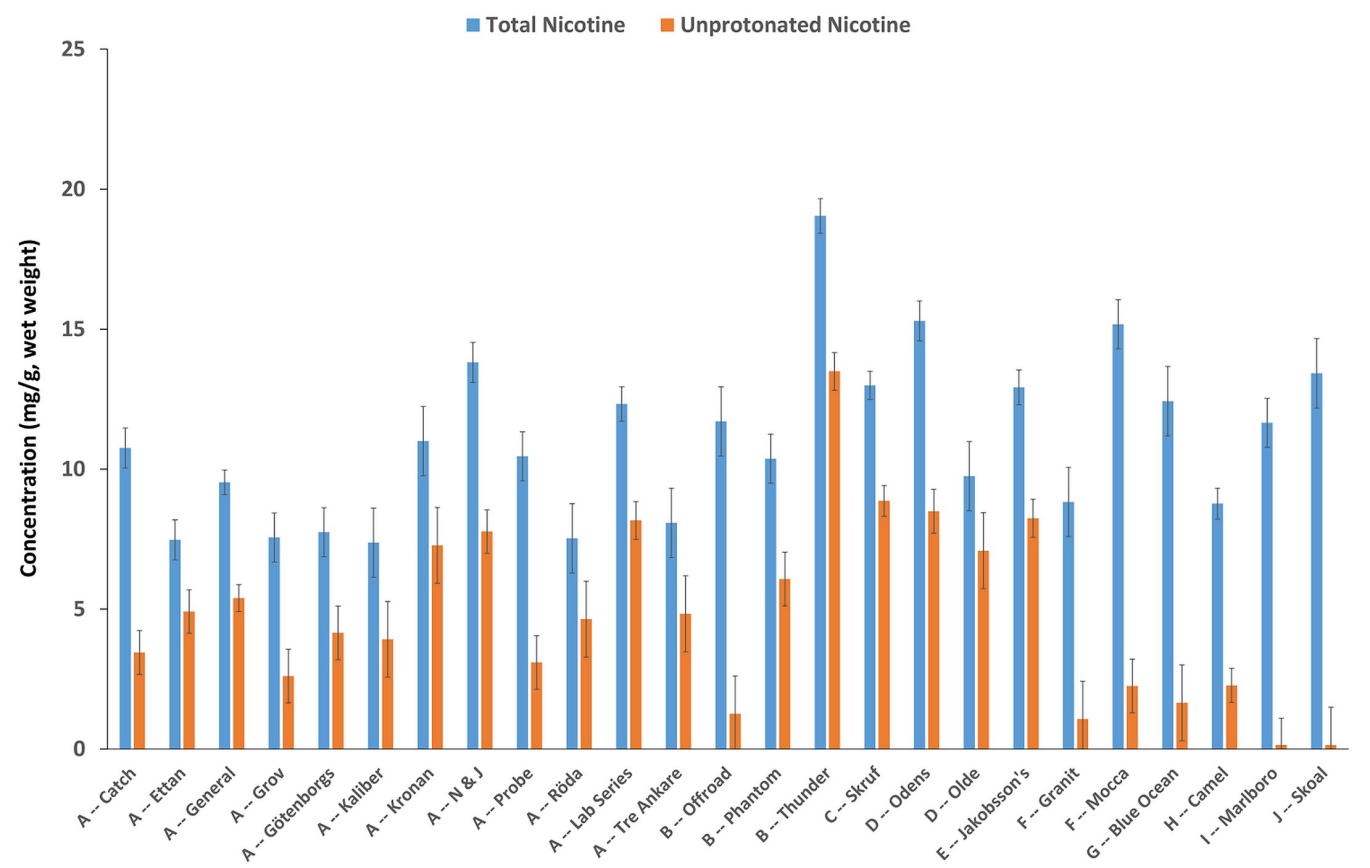
Arithmetic means of total and unprotonated nicotine found in twenty-five snus brand families made by ten manufacturers in Europe [A-G] and the US [H-J]) varied widely among all products. However, US snus had lower unprotonated nicotine compared to NE snus. All levels shown are expressed as milligrams per gram (mg/g) wet weight of product. Note. A-J represent the different manufacturers: A) Swedish Match, B) V2 Tobacco, C) Imperial Tobacco Group, D) GN Tobacco Sweden AB, E) Gotland Snus AB, F) British American Tobacco, G) AG Snus, H) RJ Reynolds, I) Philip Morris, and J) U.S. Tobacco Company. Arithmetic means and standard errors are estimates from 10,000 bootstrap resamples of the data. Error bars represent bootstrapped standard errors.

There is a wide range of total and unprotonated nicotine levels (wet wt.) across ten manufacturers and twenty-five brand families (Fig 2). A summary of all 64 snus products by manufacture and brand family is provided in the S1 Table. Swedish Match, which represents 48% of the study (12 brand families), had total nicotine ranging from 6.81 to 16.9 mg/g. Six brand families from Swedish Match products, Kaliber, Rӧda, Gӧtenborgs, and Tre Ankare among portion products, and Ettan and Grov among loose products, had the lowest arithmetic mean total nicotine concentrations, ranging from 7.37 mg/g (Kaliber) to 8.08 mg/g (Tre Ankare). V2 Tobacco, the second largest representation of snus brand families (n = 3), had total nicotine concentrations ranging from 9.47 to 20.6 mg/g, which was the widest unprotonated nicotine concentrations range (1.26−15.7 mg/g) among all manufacturers. The highest arithmetic mean total nicotine concentrations were found in the British American Tobacco Odens brand family (15.3 mg/g, SE = 0.72) and V2 Tobacco Thunder brand family (19.0 mg/g, SE = 0.62). In terms of unprotonated nicotine, the same Thunder products described above had the highest arithmetic mean unprotonated nicotine concentration (13.5 mg/g, SE = 0.68). Lastly, the lowest arithmetic unprotonated nicotine levels were found in two US brand families, Skoal (0.14 mg/g, SE = 1.35) and Marlboro (0.15 mg/g, SE = 0.96).

We compared TSNA concentrations on a wet weight basis by region and product type (Table 2). Of the five TSNAs analyzed in snus products, NNN and NAT were present in the highest concentrations. For US snus, in Table 2 –Comparison D the LS-mean concentration of NNN was 1,050 ng/g (SE = 168), and the LS-mean concentration of NAT was 804 ng/g (SE = 214). NNN and NAT levels in all NE snus were lower than in US snus, with LS-mean concentration of 615 ng/g (SE = 107) and 534 ng/g (SE = 138), respectively (Table 2 –Comparison D). In Table 2 –Comparison A, among the NE portion snus products labeled as “standard”, the LS-mean of NNN+NNK and total TSNA concentrations were 792 (SE = 129) and 1,260 (SE = 241) ng/g, wet, respectively. The LS-mean of NAT, NNK, NNN+NNK, and total TSNA concentrations were significantly higher in NE portion products with “strong” descriptors than “standard” descriptors: 733 (SE = 144), 311 (SE = 47.4), 1030 (SE = 142), and 1820 (SE = 264) ng/g, wet, respectively. The levels of NNN+NNK were significantly higher among US portion standard snus compared to NE portion standard snus (1360 [SE = 207] vs. 792 [SE = 129] ng/g wet). There was a significant difference between the LS-mean of NAB (41.9 [SE = 11.4] vs. 26.9 [SE = 12.2] ng/g wet) and NNAL (24.2 [SE = 4.17] vs. 18.3 [SE = 4.63] ng/g wet) across NE portion and loose products (Table 2 –Comparison A and C). In Table 2 –Comparison C, the LS-mean of NNN+NNK for US portion snus (1360 ng/g [SE = 207]) was significantly higher than NE portion snus (909 ng/g [SE = 128]). LS-means of NE portion, NE loose, and US portion snus for all other tobacco-specific-N’-nitrosamines were not significantly different from each other (Table 2 –Comparison C), and similarly, TSNA levels in NE snus did not differ among white and original portion varieties (Table 2 –Comparison B) or between NE All and US All categories (Table 2 –Comparison D).

**Fig 3:**
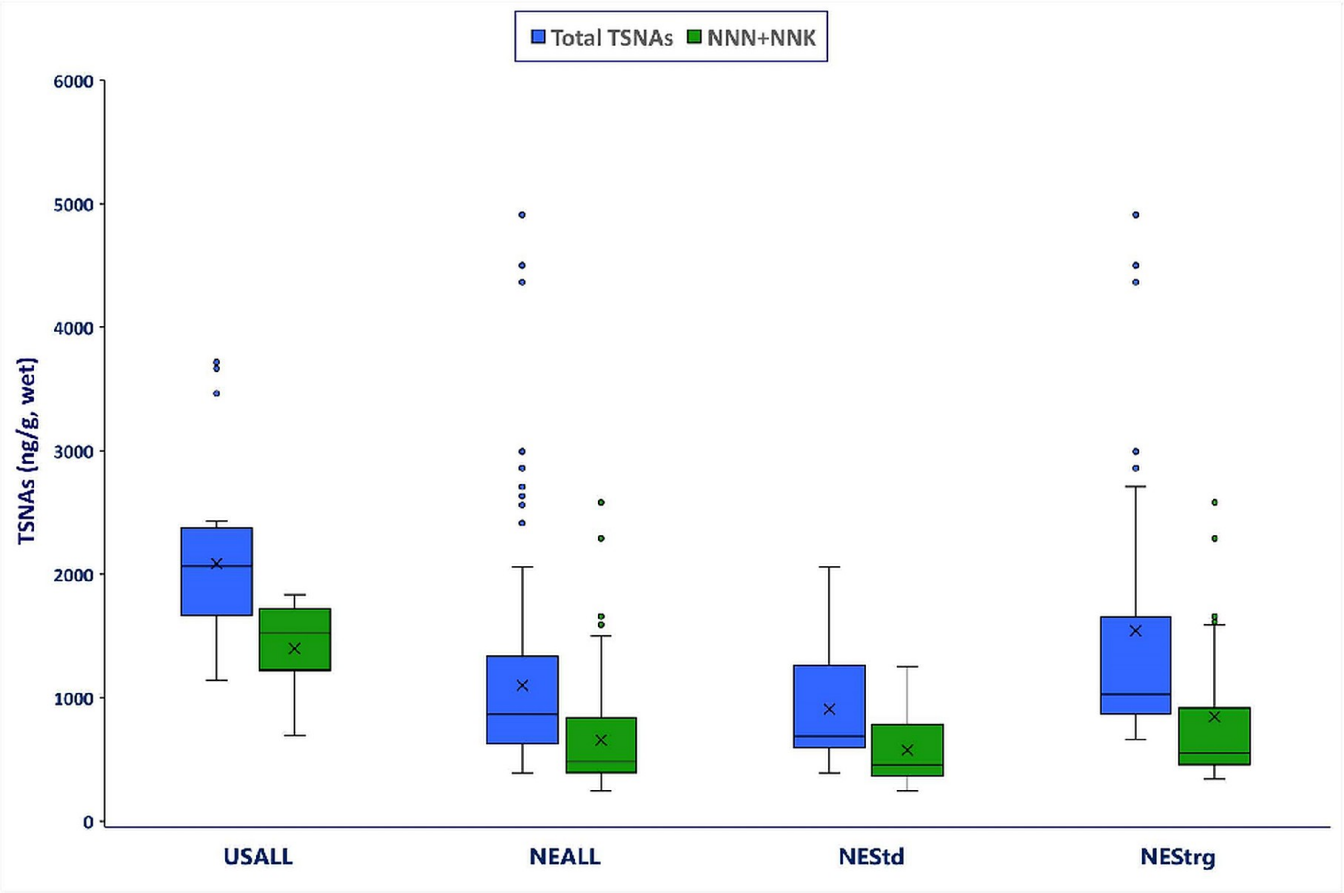
Total TSNAs and carcinogenic TSNA (NNN+NNK) concentrations (mg/g, wet weight) for US snus (USALL), Northern Europe snus (NEALL), NE snus with “standard” descriptors (NEStd) and NE snus with “strong” descriptors (NEStrg). Note: USALL (n = 8: all 8 portion); NEALL (n = 56: 7 loose and 49 portion); NEStd. (n = 39: 6 loose and 33 portion); NEStrg (n = 17: 1 loose and 16 portion).

In Fig 3, the sum of two carcinogenic nitrosamines (NNN+NNK) in US snus was approximately 2.1 times higher than in NE snus. On average, the amount of total TSNAs (NNN+NNK+NAT+NAB+NNAL) in the US snus was 2,086 ng/g, compared with 1,100 ng/g in NE snus. Moreover, there were noticeable differences between TSNA concentrations in NE loose and in portion snus varieties. On a wet weight basis, levels of NNN, NNK+NNN, NAB, and NNK were approximately two times higher in US portion (n = 8) products than in European portion (n = 49) products, 1.98, 2.01, 2.02, and 2.10 times higher, respectively. Overall, the highest NNN+NNK and total TSNA levels were detected in US portion products, followed by NE portion products, and then NE loose snus (US portion > NE portion > NE loose).

**Fig 4:**
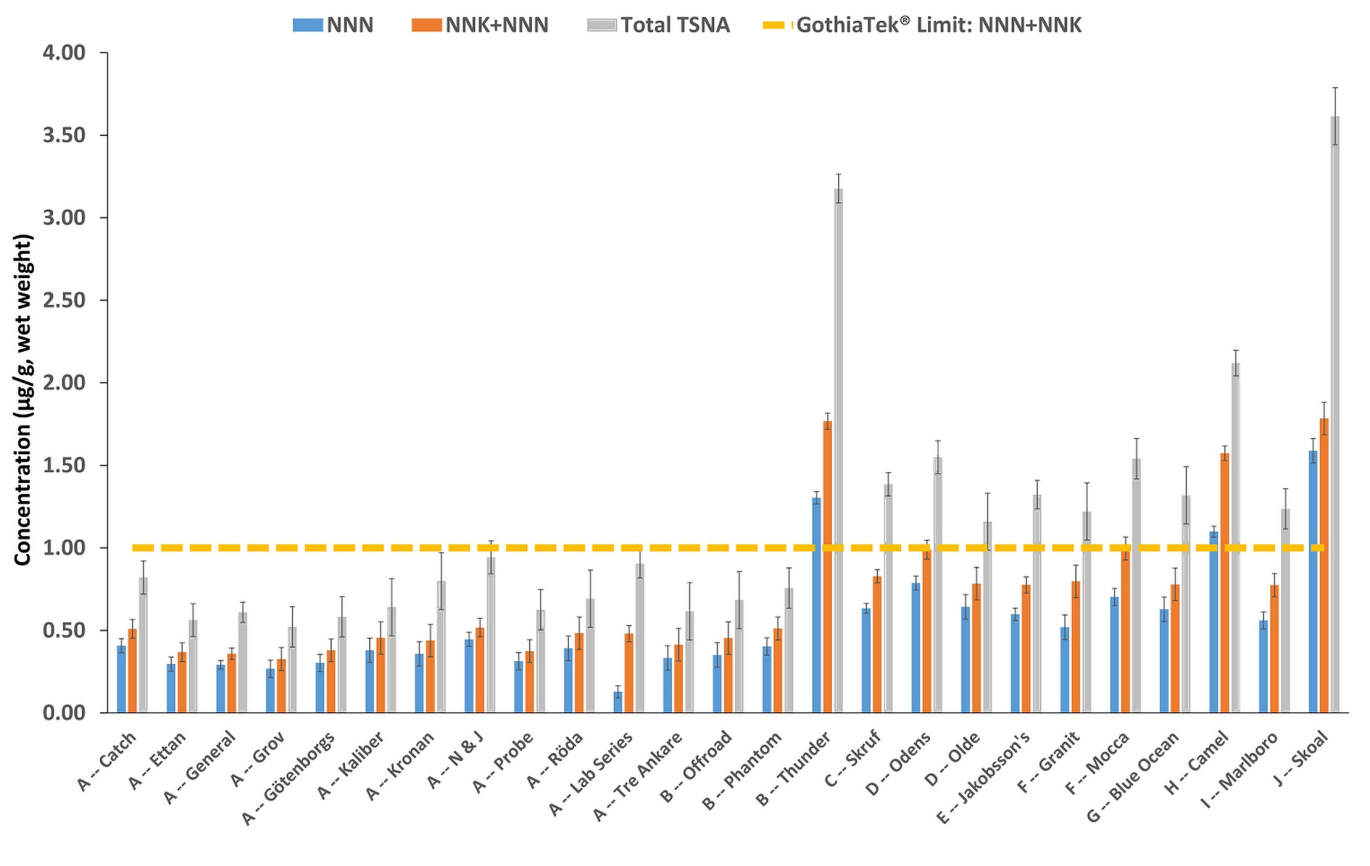
Arithmetic means comparing TSNA concentrations in snus by brand family (n = 25) and manufacturer in Europe [A-G] and the US [H-J]). Only products made by manufacturer A appears to conform to the GothiaTek® quality standard. All concentrations are expressed as micrograms per gram (μg/g) product (wet weight basis). Note. A-J represent the different manufacturers: A) Swedish Match, B) V2 Tobacco, C) Imperial Tobacco Group, D) GN Tobacco Sweden AB, E) Gotland Snus AB, F) British American Tobacco, G) AG Snus, H) RJ Reynolds, I) Philip Morris, and J) U.S. Tobacco Company. Arithmetic means and standard errors are estimates from 10,000 bootstrap resamples of the data. Error bars represent bootstrapped standard errors.

Fig 4 shows arithmetic mean NNN, NNN+NNK, and total TSNA concentrations by manufacturer on a wet weight basis. Concentrations of total TSNAs, NNN, and NNK+NNN in US snus was 1.90 to 2.13 times higher than all (portion and loose) NE snus products. All twelve Swedish Match brand families had the lowest mean concentrations of NNN+NNK (range 0.33–0.52 μg/g). The Skoal brand family (1.78 μg/g, SE = 0.10) by U.S. Smokeless Tobacco and V2 Tobacco Thunder brand family (1.77 μg/g, SE = 0.05) had the highest mean NNN+NNK concentrations.

Several minor tobacco alkaloids, which are important in TSNA formation, were measured. Anatabine (the precursor of N'-nitrosoanatabine or NAT) and nornicotine (the precursor of N'-nitrosonornicotine or NNN), which are typically seen in other tobacco products (e.g., cigarette filler), were the two most abundant alkaloids detected and identified. In US snus products, the arithmetic mean concentration of nornicotine was 244 μg/g (range 146–467 μg/g) and the arithmetic mean concentration of anatabine was 236 μg/g (range 67.6–817 μg/g). In NE snus products, the arithmetic mean concentration of nornicotine was 188 μg/g (range 118 to 433 μg/g), and the arithmetic mean concentration of anatabine was the same, 188 μg/g (range 18.3 to 561 μg/g). Snus products from both regions had concentrations of myosimine (range 4.40–56.4 μg/g), isonicotine (range 7.23–153 μg/g), and anabasine (range 21.7–145 μg/g).

Thirty-five of the 64 products analyzed in this study (55%) contained one or more of the ten flavor compounds monitored via the analytical method described above. Menthol was the most abundant flavor compound detected in both US (100%) and NE (39%) snus. Overall, menthol levels ranged from 5.61 to 11,061 μg/g. Both eucalyptol and pulegone were present in 37.5% of the US snus varieties tested. Concentrations of eucalyptol and pulegone in these US snus products ranged from 20.1–818 μg/g and 36.9–165 μg/g, respectively. Similarly, eucalyptol (27%) and pulegone (16%) were frequently seen in NE snus products with concentrations ranging from 5.24 − 288 μg/g and 6.61 − 41.7 μg/g, respectively. Methyl and ethyl salicylate were identified in one NE (General) and one US (Camel) snus brand, and diphenyl ether was detected in one NE product (Oden). Of all European snus products, a higher prevalence of flavor constituents was observed in portion products than in loose varieties.

## Discussion

Snus products vary in style, strength, and flavor. Among the portion products there is also a wide range of tobacco/portion (0.33–1.13 g). This study surveys the pH, moisture, flavors, minor alkaloids, TSNAs, and nicotine levels in 64 snus products for sale in either Northern Europe or the United States to examine similarities and differences. Our findings show some statistically significant differences exist in snus products by manufacturer, descriptor, and region. Specifically, US snus products were drier with higher TSNA levels than NE snus, while NE snus had higher pH levels and higher levels of unprotonated nicotine. Differences were also observed in other attributes of the products.

The wide assortment of additives, such as spices, oils, and flavors, found in snus may influence tobacco use among youth and adolescents [21–24]. Menthol was the most prevalent flavor constituent found in our study. Additional flavor compounds such as methyl salicylate and pulegone were also present and could contribute to the popular mint and wintergreen flavors. The portion snus style is rapidly becoming more popular than loose selections and may be targeted to younger age groups, as portion products can easily be secured in the mouth and used unnoticed [1,21].

TSNA levels were lowest in Swedish Match products that conform to the GothiaTek® quality standard. Swedish Match products clearly conformed to the GothiaTek® limits for NNN+NNK and total TSNA concentrations. However, not all NE snus made in Europe were below the threshold value for TSNAs. For instance, samples of one European V2 Tobacco Thunder brand had higher observed levels of NNN+NNK and total TSNAs compared with all other products. Additionally, NNN+NNK levels in two US snus products (Camel and Skoal) exceeded GothiaTek® criteria. While some NNN+NNK levels detected in this snus study may surpass the quality threshold, they are still well below most (non-snus) moist snuff (3.11–52.5 μg/g) and all dry snuff products (7.46–45.9 μg/g) reported in previous oral tobacco studies [11,25]. Furthermore, TSNA levels reported in snus products conforming to GothiaTek® were among the lowest in oral tobacco products [3]. For example, the snus products analyzed in this study had considerably lower mean TSNA levels than dry snuff (21.8 μg/g), moist snuff (8.69 μg/g), and chewing tobacco (3.04 μg/g) [11–12,25] and are comparable to levels found in US dissolvable tobaccos (0.50 μg/g) [3,12]. In 2016, Swedish Match further reduced the GothiaTek® levels among various toxic and carcinogenic snus constituents. For example, the average concentration of NNN+NNK is now 0.50 mg/kg [1]. Thus, self-imposed changes to certain process parameters [5] can achieve lower TSNA levels in tobacco products. These data illustrates how the manufacturing processes can minimize harmful constituents, such as TSNAs, in oral tobacco products like snus.

The lower levels of TSNAs in snus, especially products made under the GothiaTek® standard, demonstrate it is technically feasible to reduce levels of specific chemicals during the production process [5]. Processes that exclude microorganisms are highly effective in reducing TSNA levels [5–6]. However, it is not generally known how the tobacco is cleaned prior to snus manufacturing. Washing tobacco at harvest is one means of removing agricultural chemicals, microbes, and soil particles that may contain associated microbes or metals [26]. Moreover, because snus products are generally kept refrigerated at the point of sale, low product temperatures may also hinder microbial growth and generation of nitrite and TSNAs [5].

Although total nicotine levels overlapped between the two regions, pH and unprotonated nicotine varied significantly. In order to maintain a specific pH, alkaline agents like ammonium and sodium carbonates are commonly used in snus tobacco [1,5,27]. Higher product pH increases the proportion of nicotine present as unprotonated nicotine, which is more readily absorbed across membranes [28–30] and may increase product addictiveness [31–32]. Overall, unprotonated nicotine levels were highest in European portion selections, followed by European loose tobaccos, and US tobacco portion products. Among NE snus, those labeled with “strong” or “extra strong” descriptors had significantly higher unprotonated nicotine than those with standard descriptors.

The products used in this study were selected based on popularity, availability, and convenience and do not include all snus products available. This is a major limitation of this work. Additionally, we only analyzed one point of sale per brand, thus excluding shelf life variations. The sample size per region and per manufacturer also varied considerably. We were only able to find 8 US snus products at the time the study was initiated, compared with 56 NE snus products. Similarly, we only tested one brand family for a few manufacturers as opposed to 12 brand families for another. Further involving additional manufacturers and brands would help fill key information gaps.

## Conclusion

Our results identify potential key differences in the physical and chemical characteristics of snus products from the US and Northern Europe. One manufacturer consistently had products with reduced TSNA levels, likely achieved by reducing or eliminating select microorganisms, to minimize TSNA formation. The lower nitrosamine levels in select snus products clearly demonstrates that tobacco products can be manufactured with reduced carcinogens. The higher levels of unprotonated nicotine in some Swedish products, especially those designated as “strong,” was an important finding in this product survey as higher percentages of unprotonated nicotine could increase addictiveness. The higher unprotonated nicotine levels associated with certain product descriptors may be achieved through inclusion of higher nicotine tobacco sources and buffering agents [1] that increase alkalinity. Higher unprotonated nicotine levels, in turn, enhance nicotine adsorption [28] and may increase product addictiveness and contribute to adverse health outcomes linked with sustained and repetitive tobacco exposure. Future research is warranted to examine the impact of addictiveness found in reduced carcinogen exposure products, the relation of alkalinity to unprotonated nicotine, and any associated modulation.

## Supporting information

S1 Table A summary of 64 snus products (ten manufacturers [A-J] and twenty-five brand families) from the US and Northern Europe.
(DOCX)
