# Supplementary material for: Chemical analysis of snus products from the United States and northern Europe
Source: PLoS One. 2020 Jan 15;15(1):e0227837. doi: 10.1371/journal.pone.0227837 (PMC6961908; doi:10.1371/journal.pone.0227837)
Supplement: S1 Table — A summary of 64 snus products (ten manufacturers [A-J] and twenty-five brand families) from the US and Northern Europe. (DOCX) [file pone.0227837.s001.docx]

**SUPPORTING INFORMATION**

**S1 Appendix**. A summary of 64 snus products (ten manufacturers [A-J] and twenty-five brand families) from the US and Northern Europe.

|  | **Manufacturer** | **Total Number of Products** |  | **Total Number of Brand Families** | **Loose/ Standard Descriptors** | **Loose/ Strong**^1^ **Descriptors** | **Portion/ Standard Descriptors** | **Portion/ Strong Descriptors** |
| --- | --- | --- | --- | --- | --- | --- | --- | --- |
| A | Swedish Match | 31 |  | 12 | 4 | 1 | 18 | 8 |
| B | V2 Tobacco | 7 |  | 3 | 1 | 0 | 2 | 4 |
| C | Imperial Tobacco Group | 6 |  | 1 | 0 | 0 | 5 | 1 |
| D | GN Tobacco Sweden AB | 4 |  | 2 | 1 | 0 | 3 | 0 |
| E | Gotlandsnus AB | 4 |  | 1 | 0 | 0 | 1 | 3 |
| F | British American Tobacco | 3 |  | 2 | 0 | 0 | 3 | 0 |
| G | AG Snus | 1 |  | 1 | 0 | 0 | 1 | 0 |
|  | **Total: Northern European** | **56** |  | **22** | **6** | **1** | **33** | **16** |
| H | RJ Reynolds | 5 |  | 1 | 0 | 0 | 5 | 0 |
| I | Philip Morris | 2 |  | 1 | 0 | 0 | 2 | 0 |
| J | U.S. Smokeless Tobacco Company | 1 |  | 1 | 0 | 0 | 1 | 0 |
|  | **Total: US** | **8** |  | **3** | **0** | **0** | **8** | **0** |
|  | **Overall Total** | **64** |  | **25** | **6** | **1** | **41** | **16** |

^1^ Strong include label descriptors such as Strong, Extra-Strong, Stark, and Ultra-Strong
